# Measurements of roll, steering, and the far-field wake in track cycling

**DOI:** 10.1038/s41598-022-15384-3

**Published:** 2022-07-05

**Authors:** Shaun Fitzgerald, Richard Kelso, Paul Grimshaw, Andrew Warr

**Affiliations:** 1 School of Mechanical Engineering, The University of Adelaide, Adelaide, SA 5005 Australia; 2 College of Health and Life Sciences, Hamad Bin Khalifa University, Doha, Qatar; 3 Adelaide, Australia

**Keywords:** Mechanical engineering, Fluid dynamics

## Abstract

A series of measurements taken with two instrumented track bicycles in a velodrome are presented. The bicycle wheel speed, cadence, roll angle, steering angle, power, and airspeed are recorded. The experimentally-measured values are compared to existing theoretical models of roll and steering angles. The accuracy of the roll angle calculations is dependent on the fidelity of the modelled cyclist path and decreases for higher riding speeds. Experimental measurements of the steering angle show a reasonable agreement to theoretical calculations, albeit with reduced steering angles on the bends at higher speeds. There is also seen an increasing steering angle oscillation within each pedal cycle with increasing bicycle velocity which may influence a cyclist’s rolling resistance and the aerodynamic flow around the bicycle’s front end. Observations are made of changes in the flow field ahead of the bicycle due to the presence of other riders on the track, showing an effective tailwind of up to 0.7 m/s. The measured power shows a decrease at the bend entry due to the changing roll angle. Data presented in this paper provides new insights and can help to provide a validation of values used in existing track cycling analytic models.

## Introduction

Track cycling has been the subject of many mathematical models developed to predict performance or calculate a rider’s aerodynamic drag^1–5^. These models usually measure the wheel speed and power output and then, depending on the model’s complexity, calculate various other parameters to estimate either lap time or aerodynamic drag. A common feature of cycling mathematical models, primarily for track cycling, is the difference in velocity between the wheels ($V_{W}$) and the Centre of Gravity (CG) ($V_{CG}$). This difference in velocity arises from the cyclist rolling (leaning) in the bends, with roll angles often exceeding 50° for track cycling, and so the CG travels a shorter distance than the wheels, leading to a lower $V_{CG}$. As such, an accurate calculation of $V_{CG}$ requires measurement of the wheel speed and bicycle roll angle ($\phi$). Whilst the former can be measured with accuracy and ease, the latter can be more challenging to determine.

Studies comparing an experimentally-measured roll angle and a calculated roll angle have been completed by others but, to the best of the authors’ knowledge, not in a velodrome. Sanjurjo et al*.*^6^ combined the wheel speed and a 3-axis accelerometer with a custom dynamical model to measure the roll angle of a bicycle through standard riding manoeuvres. Sakai et al*.*^7^ measured the roll angle for downhill cycling with an Inertial Measurement Unit (IMU) and compared it to a model of a point mass undergoing uniform circular motion. However, both of these studies were designed for general cycling, rather than track cycling where the roll angles are much greater, the track is banked, there is a non-zero roll rate during bend transitions (which account for approximately half of the total lap), and the bicycle often follows a standard, well-defined curve. Work has also been completed for motorcycle roll angles^8^, however there can be large differences between the steering and roll angles for a motorcycle compared to a track cyclist due to the greater vehicle mass, wider tyres, and higher likelihood of the motorcycle rider displacing their centre of gravity away from the centreline of the motorcycle compared to a track cyclist.

There have been minimal efforts to measure the steering input for track cycling, which could differ from the requirements for road cycling. Restricting ourselves to pursuit events that largely follow the velodrome datum (black) line, a track cyclist will only provide minor adjustments to the steering angle as turning is generally achieved by rolling through the bends. A study by Kyle^9^ measured the steering angle around a 250 m velodrome track, showing a mean of approximately 1° on the straights, 4° on the bends and ± 2.5° with every pedal stroke. However, Kyle^9^ only provides 8.5 s of data where the exact position of the velodrome bend/straights is unclear and the experimental method is not detailed. Typical track cycling models currently ignore steering angles^3,4,10^, an exception being a forward-integration model by Fitton and Symons^5^ which uses calculations involving the track bank angle and bicycle geometry to calculate a quasi-steady-state steering angle. The steering input over a lap is not presented by Fitton and Symons^5^, with only the bend apex steering input provided over a range of speeds.

When cycling outdoors, an individual or group of cyclists create a low speed, turbulent wake behind themselves that dissipates naturally into the atmosphere^11,12^. The longitudinal distance and lateral offset behind a cyclist at which the wake becomes negligible due to natural mixing, spreading, and dissipation is largely unknown. Research has been conducted at up to 6.9 m downstream of a single cyclist for the four cardinal crank angle positions, demonstrating a lateral shifting of the wake due to asymmetry of the cyclist^12^. Union Cycliste Internationale (UCI) road time trial regulations specify a region 2 m wide and 25 m long that must be avoided to prevent drafting effects^13^. However, work by Blocken et al*.*^14^ has shown that there can be a 7% drag reduction when a cyclist is 50 m behind a motorcycle, demonstrating that the wake remains for a long distance. A cyclist would provide a smaller, but somewhat similar wake to a motorcycle. The presence of increased turbulence behind other riders in a velodrome has been previously measured in a team pursuit event, with a turbulence intensity of 16.1% measured when drafting directly behind another cyclist compared to 1.5% for a solo rider^15^. It has also been recently proposed that research should be conducted into the measurement and influence of turbulence on cycling aerodynamics^16^.

When competing inside a velodrome, the average effective distance between two opposing pursuit teams/individuals is half the lap length, generally 125 m; much further than the previously-described longest measured drafting distance of 50 m. As there is no external wind and the cyclists travel around the same path, the wake may not have enough time to dissipate before the next riders arrive, and so there can be a build-up of slow-moving, turbulent airflow that is entrained by the movement of the cyclists. The size of this wake is likely to be dependent on the number of cyclists, their speed, position on the track, and the length of the race. The smallest wake would occur in a qualification time trial where there is only a single rider on the track for a maximum of three laps. In contrast, the largest wake would occur during a bunch race where there can be up to 24 riders for up to 200 laps of a 250 m track^17^. In this scenario, the wake of each individual cyclist would merge to create a swirling flow along the track, reducing the effective head-wind and hence aerodynamic drag. The cyclists on a velodrome are not usually packed as close as in a road peloton, but this swirling flow could be compared to the inside of a peloton where the aerodynamic drag of a sheltered cyclist has been shown to be 5–10% of their isolated drag^18^. Work on speed skating has shown that skaters in an indoor skating oval can create a wind flow of 5–6 km/h along with increased mechanical turbulence^19^. This swirling tailwind flow has been mentioned before in the cycling literature^5,20^, but there has been no work completed in quantifying it as far as the authors are aware.

This work aims to simultaneously measure the roll angle, steering angle, power, airspeed and wheelspeed of a bicycle to provide insight into the movement of a track cyclist in a velodrome. This can be used to provide guidance in competitive track cycling or further research on aerodynamics and bicycle dynamics on banked tracks.

## Results

**Figure 1:**
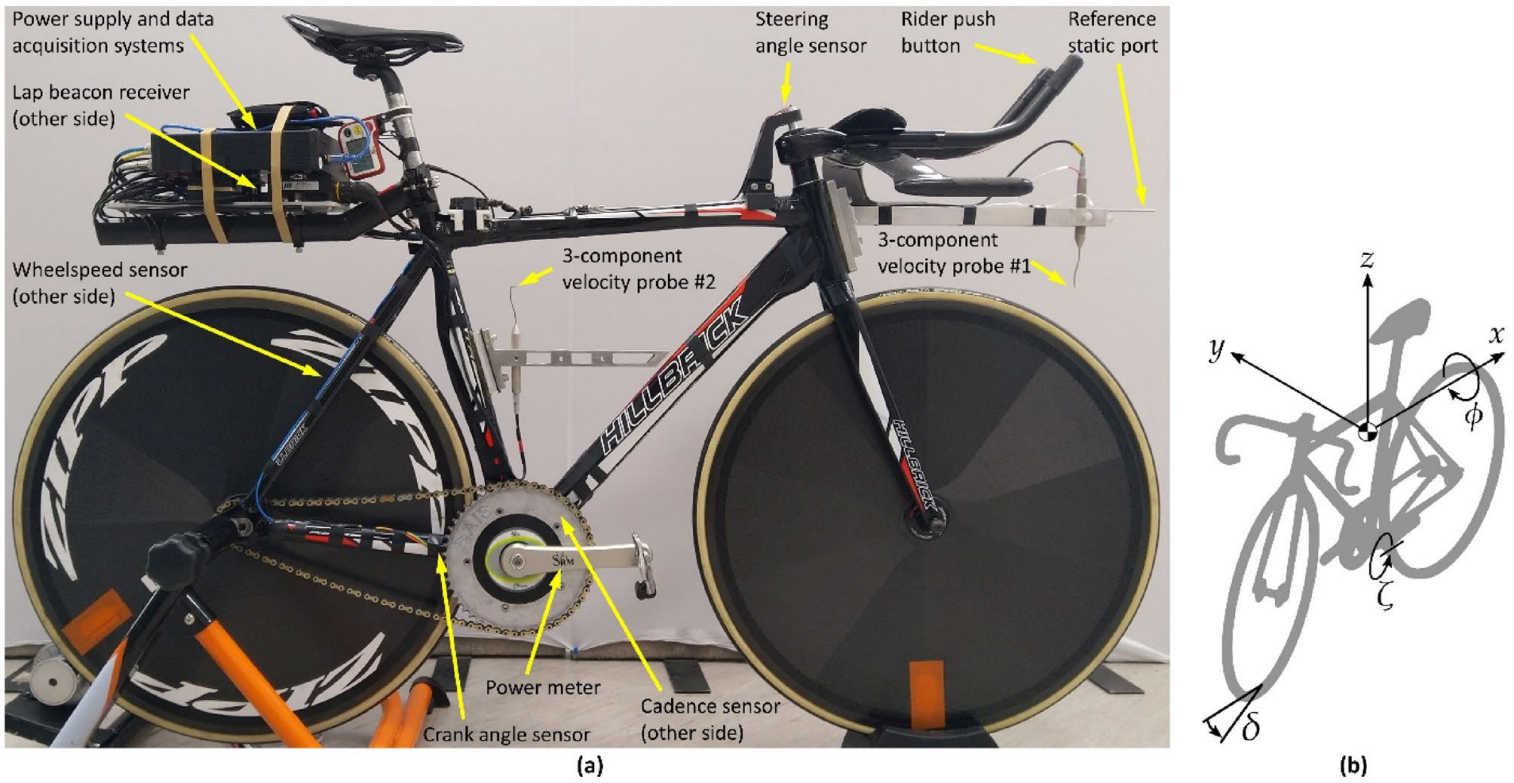
(**a**) Primary bicycle used for testing and (**b**) bicycle coordinate system.

Experimental testing was completed with an instrumented bicycle that recorded the airflow velocity anterior to the head tube, the wheel speed, (pedalling) cadence, power, crank angle and steering angle (Fig. 1a)^21^. A second bicycle measured wheel speed, cadence, power, and roll angle. The bicycle-fixed coordinate system has the x-axis directed to the bicycle’s rear, the y-axis to the bicycle’s right, and the z-axis upwards. This orientation has a positive roll angle ($\phi$) when the cyclist is leaning left and a positive steering angle ($\delta$) when the cyclist is steering left. The crank angle ($\zeta$) is defined as zero with the cranks horizontal and the right crank forwards. A diagram of the coordinate orientations are shown in Fig. 1b. For this work, the bicycle with the aerodynamic and steering sensors will be defined as the primary bicycle, and the bicycle with the roll sensor defined as the standard bicycle. Two elite cyclists rode the two separate bicycles for all tests and were paced by their coach to maintain a consistent, self-selected lap time. Full details of the setup are provided in the Methods section.

### Theoretical calculations

The measured results were compared to standard theoretical calculations of roll and steering used in previous track cycling models. To do so, a custom geometric model of the 250 m velodrome datum line was defined with reference to theodolite measurements of the physical line. This track model consists of two straight segments, two circular arcs in the middle of the bends, and four transition curves connecting the straights and the bends. The transition curves were designed with the curvature increasing from zero in the straights to 0.0465 m^−1^ in the circular bends by following a half sine wave trough-to-peak path^22^. All theoretical calculations assume that the cyclist follows the datum line exactly and all laps start at the pursuit line in the middle of the home straight and are left-turning.

By assuming the cyclist/bicycle system is a point mass and equating moments about the tyre/track contact point, the roll angle can be calculated from the wheel speed and track geometry with^4,10,22^:

1 $$ \tan \phi = \frac{{\kappa V_{W}^{2} }}{g}\left( {1 - h_{CG} \kappa \sin \phi } \right). $$

Here $\kappa$ is the turn curvature, defined as the inverse of the turn radius, $h_{CG}$ is the height of the centre of gravity of the bicycle/rider, and $g$ is the acceleration due to gravity. This is a transcendental equation that requires iteration to solve, but it converges to within 99% of the final result within three iterations with the Newton–Raphson method. Once the roll angle is found, $V_{CG}$ can be calculated with:

2 $$ V_{CG} = V_{W} \left( {1 - h_{CG} \kappa \sin \phi } \right). $$

Theoretical steering input calculations were undertaken following the method outlined by Fitton and Symons^5^ with wheel cornering and camber stiffness coefficients obtained from Doria and Roa^23^.

### Changes over a lap

**Figure 2:**
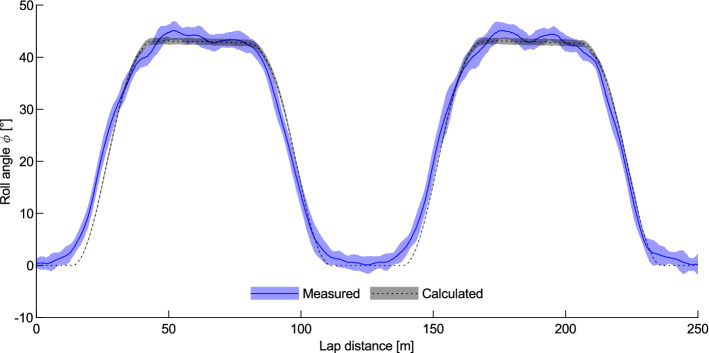
Measured and calculated roll angle averaged over 17 laps with a mean wheel speed of 14.0 m/s. The shaded area indicates ± 1 standard deviation.

The roll angle results from the standard bicycle are shown in Fig. 2 alongside calculated roll angles from Eq. (1). There can be seen a continuous change in the measured roll angle from the straights to the bends along with some small variations in the middle of the bends. The calculated roll increases at a steep rate during the transition zone, following the increase in track curvature during the bend transition, then remains constant through the primary curve of the bend. The lap-averaged root mean square (RMS) error between the measured and calculated results is 2.1°. The most significant discrepancy between the calculated and measured results occurs at the start and end of the track transitions and is likely due to the uncertainty of the modelled cyclist’s path. A cyclist will not follow the datum line exactly, rather they can ride outside the datum line, higher up the bank on the straights, and then close to the datum line in the bends, creating a discrepancy between the actual turn radius of the wheels and the expected turn radius based on the datum line. This is done so that the cyclist can better control their change in roll from the straights to the transitions and onto the bends, lessening the maximum roll rate by increasing roll early and decreasing roll later. This pattern could also be due to the rider maintaining a constant height across a lap as the datum line increases slightly in elevation in the bends. In addition to the influence of lap position, there is also a secondary variation in roll due to the roll of the bicycle within each pedal cycle^24^.

**Figure 3:**
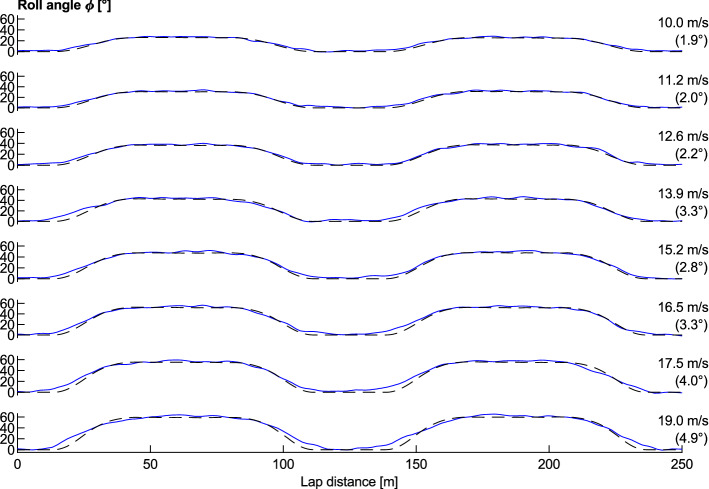
Measured (solid blue lines) and calculated (dashed black lines) roll angle for a range of wheel speeds. Shown on the right is the mean wheel speed and RMS error between the measured and calculated roll angles. Data is averaged over two laps for each speed.

A test was conducted by riding the standard bicycle behind a derny (motorised pacing bicycle) for two laps at a constant speed and then accelerating to a higher speed for further laps. The use of a derny facilitated steady speed pacing and allowed higher velocities than was achievable by a solo rider. The results for the roll angle across various speeds are shown in Fig. 3. At low speeds, the cyclist can follow the datum line closely and so the calculated curve closely matches the experimental results, whilst at higher speeds the rider was observed to travel wider on the straights and closer to the datum line in the middle of the bend. This is confirmed by tracking the wheel distance travelled over each lap, which increases from 252.0 m at 10 m/s to 253.8 m at 19 m/s, as well as the increasing lap-based RMS error between the measured and calculated roll angles, shown in brackets on the right of Fig. 3. The combination of increasing wheel distance travelled, increasing RMS error, and increasing discrepancy between the measured and calculated values of roll angle around the change between the straights and transitions indicates a more circular trajectory at higher speeds.

**Figure 4:**
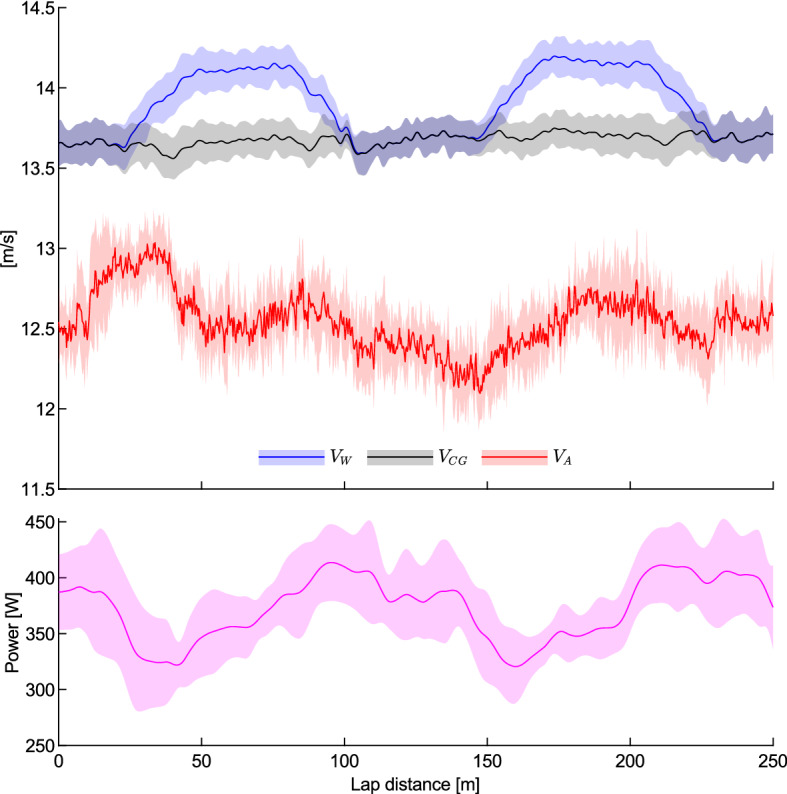
Measured wheel speed, calculated centre of gravity speed, measured air speed, and measured power averaged over seven laps with a mean wheel speed of 13.9 m/s. The shaded area indicates ± 1 standard deviation.

The measured wheel speed ($V_{W}$), calculated centre of gravity speed ($V_{CG}$, Eq. (2)), and measured airspeed ($V_{A}$), averaged over seven laps, are shown in Fig. 4. On a small scale there is a random fluctuation due to inter-lap variations (as seen in Fitzgerald et al*.*^15^) that is present due to the averaging over just seven laps, but on a larger scale some clear patterns emerge. There is a distinctive increase in $V_{W}$ in the bends whilst $V_{CG}$ remains relatively constant throughout each lap. This shows that the rider attempted to maintain a constant $V_{CG}$ in order to maintain a constant lap time, and to accommodate this they increased their cadence in the bends and decreased it in the straights. The increasing cadence corresponds to a drop in their power output early in the bends that then steadily increases as they exit towards the straights. The velocity of the air is always less than $V_{CG}$, primarily due to the upstream effect of the cyclist and bicycle slowing down the air at the measurement probe tip^15,18^.

**Figure 5:**
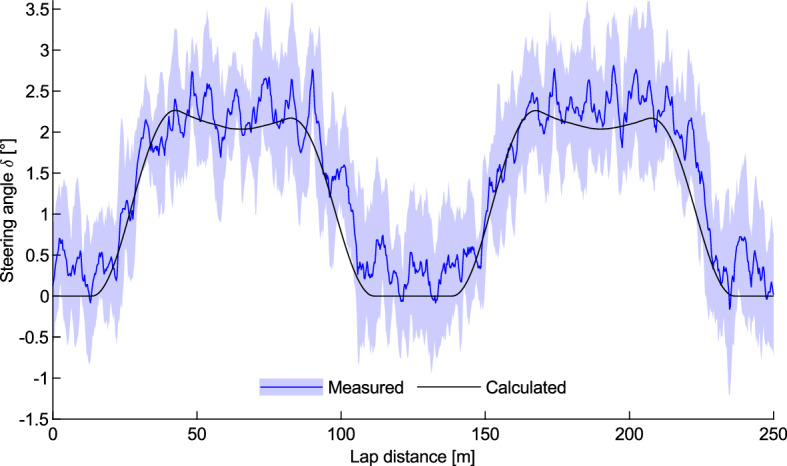
Measured and calculated steering input angle averaged over seven laps with a mean wheel speed of 13.9 m/s. The shaded area indicates ± 1 standard deviation.

The measured and calculated steering input angle over a single lap is shown in Fig. 5, displaying a distinctive variation with position on the track. There is a good agreement between the experimental and calculated steering angle values apart from scatter due to the cadence-frequency roll, steering of the cyclist during each pedal cycle, and general random variation. There is a steady increase during the bend transition, leading to an approximately-constant steering angle throughout the bend. The twin peaks observed in the calculated steering angle are due to the constant turn radius but varying bank angle causing a change in the steering angle. The higher first peak is due to the shallower bank angle on the bend entry compared to the bend exit. This minor change in the steering angle in not seen in the experimentally-measured values as it is within the variations due to other factors not accounted for in the calculations.

**Figure 6:**
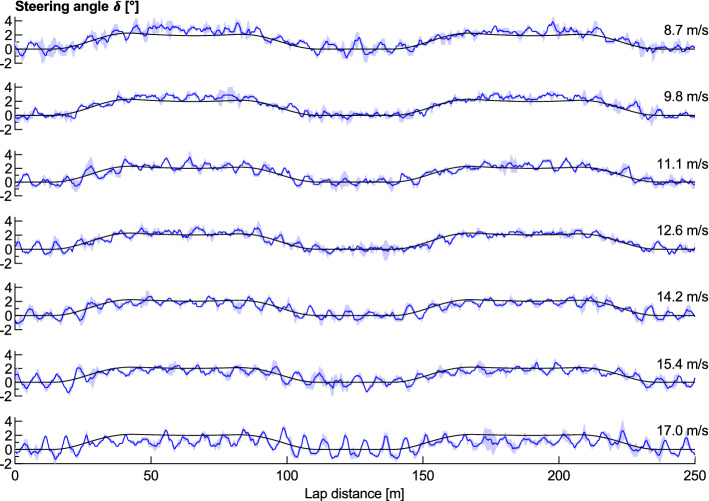
Measured (blue) and calculated (black) steering input angle for a range of wheel speeds. Averaged over two laps for each speed.

The magnitude of the required steering angle in the bends is dependent on the cyclist’s speed, as shown in Fig. 6 for the bicycle ridden at a series of controlled speeds behind a derny. The measured steering angle along the straights was consistent for all speeds; however, it steadily decreased during the bends with increasing speeds. This differs from the results for steering by Fitton and Symons^5^ who calculated an increase in steering angle with speed until the speed at which the cyclist will roll perpendicular to the bank, after which the steering angle would decrease. This theoretical speed for the present velodrome is 14 m/s at the bend apex. Fitton and Symons^5^ did not provide any experimental validation of their calculations. Furthermore, the measured steering angle values were generally greater than the calculated values for low speeds but less than the calculated values for high speeds.

### Changes over a crank cycle

**Figure 7:**
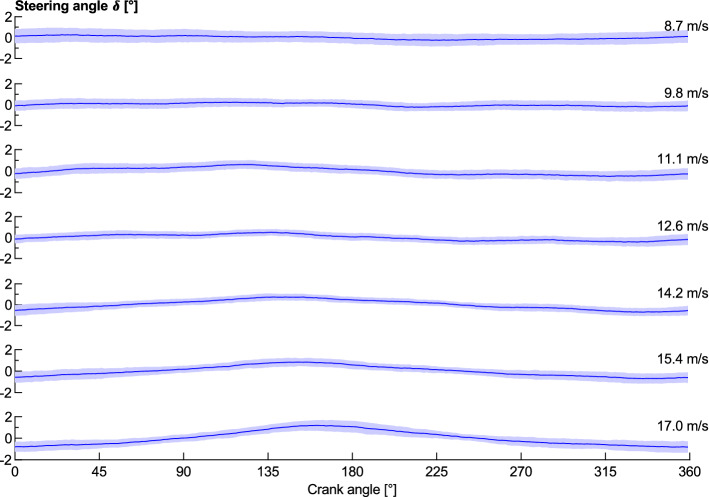
Measured steering angle over each crank cycle for a range of wheel speeds. The shaded area indicates ± 1 standard deviation. Averaged over two laps for each speed.

There is a cyclic fluctuation in the steering angle within each pedal cycle, where the rider is found to oscillate the front wheel side to side. The magnitude of this steering angle fluctuation increases with the cyclist’s speed, as shown in Fig. 7. This figure’s data has had the mean value of each pedal cycle subtracted to isolate it from the effects of lap position. The results show that there is minimal fluctuation in steering input within a crank cycle at low speeds, whilst at higher speeds there is an increasing oscillation with a maximum value at approximately 160° to 170°. This indicates that the cyclist will turn the front wheel and handlebars in the direction of the leg currently producing the power stroke. The steering angle is seen to oscillate ± 1.5° through each pedal stroke at 17 m/s, less than the ± 2.5° observed by Kyle^9^ who did not mention at what speed it was recorded. This oscillation can be expected to increase the tyre rolling resistance by creating increased tyre scrubbing, and also change the yaw angle of the airflow incident on the front wheel, thereby influencing the direction of flow and turbulence over the wheel and rest of the bicycle.

### Turbulence and wakes

A specific test was conducted during a training session warm-up where there was a group of 15 cyclists riding in a bunch around the velodrome and the primary bicycle was ridden approximately half a lap behind. A second test was conducted with just the primary bicycle ridden solo around the empty track after the velodrome airflow had been allowed to settle for 15 min. These two tests were intended to determine if there was any build-up in the wake of a cyclist that circulates the velodrome, and to measure changes in turbulence.

A key measure of the air flow relative to the bicycle/rider is the normalised airspeed, $K = V_{A} /V_{W}$, which measures the ratio between the airspeed and the wheel speed and can be used to indicate a tail or head wind. Another measure is the turbulence intensity, which was calculated in the three orthogonal directions as well as the total component. Intensity calculations were performed with Reynolds decomposition^25^ based on fluctuations above the velocity signal filtered with a Savitzky-Golay filter with a window size equal to the mean cadence period, as detailed in Fitzgerald et al*.*^15^ Integral length scales of turbulence over each lap were also calculated based on velocity fluctuations in the three orthogonal directions following Emes et al*.*^26^. Results were averaged over 6 laps for both the empty-track and warm-up tests and are shown in Table 1.

**Table 1:** Comparison of velocity and turbulence measurements for the empty track test (1 cyclist) and the warm-up test (16 total cyclists on the track), averaged over 6 laps for each test at 13.8 m/s.

	Empty track	Warm-up test
$K$	0.902	0.851
$I_{uvw}$ (%)	1.06	2.61
$I_{uu}$ (%)	1.28	2.60
$I_{vv}$ (%)	1.00	2.81
$I_{ww}$ (%)	0.87	2.41
$L_{u}^{x}$ (m)	8.26	6.18
$L_{v}^{x}$ (m)	11.05	6.73
$L_{w}^{x}$ (m)	6.97	1.29

The values for the normalised airspeed are < 1 for both tests due to the upstream deceleration of the airflow ahead of the rider and bicycle^15,18^ and the bends reducing the airspeed relative to the wheel speed. Between the warm-up and empty track tests there is a velocity ratio reduction of 0.05 at 13.8 m/s, representing an effective tail-wind of 0.7 m/s. This tailwind is likely to be in the form of a swirling flow that follows the riders around the track and builds up over the course of several laps due to entrainment of the wake. The air speed of 0.7 m/s is a low absolute value, just above the minimum observable speed for a standard hand-held wind anemometer^27^, and so is unlikely to be clearly observed by typical track-side stationary measurement.

Considering just the power required to overcome aerodynamic drag (ignoring all other energy losses such as rolling resistance), the relative speed increase achieved with a velocity ratio of $K$ is given by

3 $$ \frac{V\left( K \right)}{{V\left( {K = 1} \right)}} = K^{ - 2/3} . $$

So, for a tailwind equal to 5% of their speed ($K = 0.95$), a cyclist can increase their speed by approximately 3.5% with the same power output. This simple equation is a best-case scenario, but it provides a helpful indication that becomes more accurate at higher speeds.

In addition to the change in airspeed, there is also an increase in the turbulence when riding in the wake of a series of riders. When riding half a lap behind the other cyclists, the turbulence intensity of the streamwise flow component approximately doubles, whereas the spanwise and vertical components triple relative to their empty track values which indicates further mixing of the flow in all directions. The ratios $I_{uu} /I_{vv}$, $I_{uu} /I_{ww}$, and $I_{vv} /I_{ww}$ also converge closer to 1, as the flow becomes more ‘normalised’ to isotropic turbulence. Furthermore, the integral length scale of the streamwise component is reduced by 25%, spanwise component by 39%, and vertical component by 81% relative to empty track values. This demonstrates that the turbulence decays more rapidly, caused by the break-up of larger turbulent eddies. When riding solo the larger turbulent eddies take longer to decay, and so larger length scales are recorded.

## Discussion

There is a good agreement between the measured and calculated roll angle for an isolated cyclist. This is desirable as it demonstrates that with just a measure of wheel speed and an accurate model of the velodrome track geometry, the roll angle can be determined for an individual pursuit with a lap-averaged RMS error range of 1.9° to 4.9°. Several models used to predict lap times or calculate aerodynamic drag require this calculation^3–5,10,28^ but little work has been done verifying this intermediate result. The accuracy of the roll angle calculations is highly dependent on the accuracy of the modelled bicycle turn curvature and it decreases with higher speeds as the cyclist will tend to deviate further from the velodrome datum line and ride wider in the straights, reducing the roll rate. Using an adjustable track geometry model^22^, it would be possible to replicate this deviation by creating an artificial cyclist path with a wider track span, an increased lap length, and an identical circular arc bend. This keeps the bend apex in the same position but shortens the straights and extends the bend transition length to simulate a cyclist riding wide along the straights.

The calculated velocity of the centre of gravity was shown to be relatively constant throughout the lap when the cyclist was instructed to maintain a constant lap time. This is in contrast to the wheel speed which showed a substantial increase in the bends, and the power which decreased at the start of the bends before steadily increasing as the cyclist exited the bends. This variation in power has been previously observed by Craig and Norton^29^ who specify a minimum power output at the point of highest velocity in the bends for a 4000 m individual pursuit. The reason for this power decrease in a bend is unclear from a simple overview of the typical track cycling power demand model^3–5,10^. The aerodynamic drag should remain relatively constant through a bend as $V_{A}$ can be reasonably approximated as $V_{CG}$, with a minor variation from the influence of cornering flow^15^. In contrast, the power demand from rolling resistance is likely to increase due to the increased wheel speed and centrifugal force. The decrease in power is thus likely to be due to changes in kinetic and potential energy from the motion of the centre of gravity through the transitions. When leaning into a bend, there is a decrease in potential energy due to the lower centre of gravity, which is transformed into an increase in kinetic energy. The cyclist will accordingly reduce their power output at the bend entry and then increase it through the bend exit. Fitton and Symons^5^ include this change in potential energy in their mathematical model as both a positive and negative influence on power. This change in power between the bends and the straights is also likely to be dependent on the rider, with different athletes riding with different styles. The causes of the change in power between the straights and the bends should be an area for further research.

This work presents measured and calculated steering angles around a velodrome track, showing a steering angle of approximately 2° in the bends and up to a ± 1.5° oscillation within each pedal cycle. The results show a decreasing bend steering angle and increasing steering oscillation with increasing wheel speed. The decreasing bend steering angle with increasing speed differs to previous theoretical calcuations^5^ and is likely due to an increasing deviation from the datum line and the cyclist taking a more circular trajectory. It could also be due to the simplified, linear theoretical steering calculations not fully encompassing the complete non-linear bicycle dynamics. These observations are important as a track cyclist experiences increased scrubbing resistance when the front wheel is turned away from the direction of travel^3,9^. The change in steering angle can also influence the airflow around the front of the bicycle, inducing a further airflow yaw angle and changing the aerodynamic profile of the bicycle front^21^.

The increased tailwind observed with other riders on the track is a feature that has been commented on in prior work, but to date only anecdotal evidence has been supplied. Fitton et al*.*^30^ measured an un-specified speed of airflow tangential to the direction of motion of a team pursuit squad. Spoelstra et al*.*^31^ describe a “systematic tailwind” from the motion of a single cyclist in a closed hall. D'Auteuil et al*.*^19^ describe this flow entrainment on speed skating ovals where airspeeds up to 1.6 m/s were postulated. This artificial tailwind can reduce the aerodynamic drag experienced by a cyclist; as an example, the earlier suggested speed increase of 3.5% results in an 8.4 s time advantage over a 4 min race. However, if they remain half a lap apart, this tailwind is likely to impact all competitors on the track equally.

The increasing turbulence observed when behind a group of riders, even when they are 125 m ahead of a cyclist, is an important result as the majority of cycling aerodynamic research is conducted using low-turbulence wind tunnels or computational fluid dynamics models^12,15,16,20^. Turbulence properties can change the characteristics of the flow around a rider, particularly the initiation of laminar-to-turbulent transition in the boundary layer, which dictates the occurrence of the drag crisis^32–34^. It has been previously shown for speed skating, which operates in a very similar Reynolds number range to track cycling, that replicating on-track turbulence in a wind tunnel can induce the drag crisis at a lower wind speed compared to low-turbulence results^19^. Further work on large commercial vehicles has also shown the importance of replicating the real-world turbulence for aerodynamic research^35,36^. This has an implication for cycling research as the primary method for drag reduction of the rider is through the use of artificial roughness to induce the drag crisis^20,37^. If there is an increased level of atmospheric turbulence, then the drag crisis could be naturally induced on bluff body components and the addition of artificial roughness will increase the skin friction without any corresponding reduction in the pressure drag, increasing the total drag rather than reducing it. Detailed investigations into the effects of freestream turbulence on cycling should be an area for future research.

## Conclusions

This paper outlines a series of experimental measurements from an instrumented track bicycle in a velodrome. The roll angle is measured and compared to existing theoretical calculations, showing good agreement when the cyclist follows the track datum line, confirming the applicability of quasi-steady dynamical models to pursuit cycling. Measurements of the steering angle show reasonable agreement with calculated values and show an approximately 2° turn in the bends. When riding at higher speeds, the cyclist is seen to take a more circular trajectory to extend the bend transition and reduce the roll rate, and the steering angle oscillation over each pedal cycle is found to increase. The measured power shows a higher value in the straights and a decrease at the bend entry where there is a conversion between potential and kinetic energy due to the change in roll angle. There is also a change in the airflow when other riders are present, creating a swirling flow around the track, reducing the apparent headwind and increasing the freestream turbulence experienced by an isolated cyclist. This swirling and turbulent flow could have an impact on potential drag reduction techniques for track cycling.

## Methods

### Instrumented bicycles

Testing was completed with the primary bicycle (55 cm Top Tube, 52 cm Seat Tube, Hillbrick Pista, Australia) shown in Fig. 1a. Two 4-hole pressure probes (Cobra, TFI, Australia), with the first located anterior to the head-tube and the second between the rider’s legs, provides 3 components of velocity at a 2500 Hz sampling rate. Data from the middle probe (#2 in Fig. 1a) is not presented here and can be found in Fitzgerald et al*.*^21^. Magnetic reed switches on the rear wheel and the cranks provide wheel speed and pedalling cadence, respectively. An optical switch measures the crank angle with a measurement tolerance of 360°/53 = 6.8° using the method detailed in Fitzgerald et al*.*^21^. A push-button placed on the handlebars is used for the rider to signal particular events and for time synchronisation between different Data Acquisition Systems (DAQ). A non-contact rotating analogue rotary position sensor is mounted to the top of the steering axis and records the steering angle with a 0.4° resolution at a 500 Hz sampling frequency. Two beacon transmitters placed at either pursuit line in the middle of the straights were detected by a receiver mounted on the bicycle, providing accurate times of every half-lap. A crank-based power meter recorded the rider’s input power and cadence (Schoberer Rad Meßtechnik (SRM), Germany). The aerodynamic data, wheel speed, cadence, crank angle, and push button signals were recorded by one DAQ, (TFI, Australia), whilst the steering angle, beacon receiver, cadence, and push button signals were recorded by a second DAQ (Pi Research, United Kingdom), and the power and cadence by a third (SRM PowerControl V). The same signals from the cadence sensor and push button were recorded by all DAQs for common-time synchronisation.

A second track bicycle (54 cm Cervélo T4, Canada), known as the standard bicycle, had a 9-axis Inertial Measurement Unit (IMU), including a 3D accelerometer, 3D gyroscope and 3D magnetometer, firmly mounted posterior to the seat tube. The IMU sensor outputs were fused with a commercial Kalman filtering system (Xsens Kalman Filter) to calculate 3D orientation measurements. This bicycle had a crank-based SRM power meter, cadence sensor and a five-magnet wheel speed sensor for improved speed resolution. Lap timing was recorded with a beacon receiver which detected the transmitters placed at both pursuit lines. All data was simultaneously logged with a single custom-built DAQ system. The standard bicycle does not have the bulky pannier rack or probe holders and so doesn’t have the capacity to record the aerodynamic data, crank angle, or steering angle. However, this DAQ system is small and lightweight and so has a negligible influence on the aerodynamics or mass distribution of the bicycle, permitting it to be ridden in a ‘race ready’ state. This enabled testing of the roll angle at higher speeds.

The pressure probe has a manufacturer-specified accuracy of ± 0.3 m/s and ± 1° in yaw and pitch for speeds of 2–30 m/s in a ± 45° cone, with a frequency response of 600 Hz and 16 bit digital resolution. The probe was aligned to the bicycle frame at the velodrome track with a custom-built alignment fan with an estimated alignment error of ± 0.5° in yaw and pitch angles. The IMU has an angular resolution of 0.05°, dynamic accuracy of 2°, and a 100 Hz sampling rate. The power meter has 8 strain gauges with an accuracy of ± 0.5%.

The bicycles were ridden by two elite male cyclists who provided informed consent before participating. All experimental methods were performed according to relevant guidelines and regulations, and the procedures were approved by the University of Adelaide Human Research Ethics Committee (H-2019-018). The rider’s lap times were paced by their coach with instructions to maintain a speed that provided as consistent a lap time as possible. The cyclist maintained a time-trial position on the bicycles and no instructions on how to ride were provided. Testing behind the derny involved two laps at a consistent speed interspaced with an accelerating lap to achieve the next consistent speed.

### Velodrome

Testing was conducted at the Adelaide SuperDrome with a 250 m lap length as defined by the black datum line marked on the track surface. The track geometry was measured by a theodolite survey, providing detailed 3D coordinates of the velodrome surface. A custom mathematical model with an apex turn radius of 21.5 m and track span of 45 m was fitted to the measured geometric data, with the method fully detailed in Fitzgerald et al*.*^22^. This model has *G*^3^ geometric continuity across the lap, providing continuous acceleration and roll rate. There is no change in elevation of the modelled datum line. Measurements of the bank angle were taken from the theodolite data and checked with an inclinometer.

### Considerations for further work

One of the errors in this testing is that the rider does not always exactly follow the datum line; rather they will generally ride high in the straights and then come in close around the bends. There is also a certain level of variability in the bicycle path which can serve to increase the ridden distance compared to the official lap distance. The extent of this discrepancy can be estimated by calculating the ridden distance around the track from the wheel speed sensor, and it was found that the rider rode an extra 2.8 ± 0.67 m (mean ± standard deviation) each lap. This will impact the true turn radius at each position around the velodrome, influencing theoretical calculations of $\phi$, $V_{CG}$, and $\delta$. The magnitude of this error is influenced by the skill and experience of the cyclist, with professional athletes trained to ride with a steady pace and little deviation from the datum line.

The cyclist and bicycle are modelled as a combined point mass at the centre of gravity in the symmetry plane, ignoring the influence of any features such as bicycle geometry, gyroscopic effects, movement of the cyclist/bicycle due to pedalling, or general movement of the cyclist. The calculations assume that the steering input is small and thus does not influence calculations of roll^5^. In addition, lateral aerodynamic forces due to curved aerodynamic flow and turning of the front wheel are not considered, which could have an impact on both roll and steering angles. The results presented are for a steady-state lap where large accelerations, such as occur in sprinting, do not have an influence.

Considering a variety of distinct cyclists instead of only one would enrich the analysis on roll and steering angle. Different cyclists may have different styles of riding and follow different paths around the velodrome, which could influence roll angles, power output, and steering angles. This was a pilot study to observe the major effects, and future work could involve the testing of a variety of cyclists to achieve a more representative observation of the general professional cycling population. Additionally, this experiment has been conducted at only one velodrome, and different velodromes have different geometries, so these results may not be representative of all velodromes. However, the overall geometries of most 250 m velodromes are very similar, having small differences between straight lengths and bend radii, with the largest differences being with banking styles^22^. With an accurate model of the track geometry for a particular velodrome, the overall results presented here could be replicated at any velodrome.

## Data Availability

All data generated or analysed during this study are included in this published article.
